# Patient-reported outcomes and measures applied in individuals undergoing colonoscopy surveillance for colorectal cancer: a scoping review

**DOI:** 10.1007/s11136-026-04270-4

**Published:** 2026-06-06

**Authors:** Wudneh Simegn Belay, Erin L. Symonds, Melkalem Mamuye Azanaw, Meseret Derbew Molla, Molla M. Wassie, Geraldine Laven-Law, Martha A. Menberu, Muktar Ahmed, Billingsley Kaambwa, Norma B. Bulamu

**Affiliations:** 1 Flinders University, College of Medicine and Public Health, Flinders Health and Medical Research Institute, Adelaide, South Australia Australia; 2 Department of Social and Administrative Pharmacy, School of Pharmacy, College of Medicine and Health Sciences, University of Gondar, Gondar, Ethiopia; 3 Gastroenterology Department, Flinders Medical Centre, Southern Adelaide Local Health Network, Bedford Park, South Australia Australia; 4 Department of Public Health, College of Health Sciences, Debre Tabor University, Debre Tabor, Ethiopia; 5 Department of Biochemistry, School of Medicine, College of Medicine and Health Sciences, University of Gondar, Gondar, Ethiopia; 6 South Australian Immunogenomics Cancer Institute, Adelaide University, Adelaide, South Australia Australia; 7 College of Health, Adelaide University, Adelaide, South Australia Australia

**Keywords:** Elevated risk population, Colorectal cancer, Colonoscopy surveillance, Patient-reported outcomes, Patient-reported outcome measures

## Abstract

**Purpose:**

Clinical guidelines recommend regular colonoscopy surveillance for individuals at elevated risk for colorectal cancer (CRC). While colonoscopy surveillance is proven to reduce the incidence of CRC, colonoscopy is an invasive procedure that can impact patient-reported outcomes (PROs). Assessment of PROs is recommended as a key indicator of the quality of health service delivery. However, there is no standard set of PROs and PRO measures (PROMs) to be applied in individuals undergoing regular surveillance colonoscopy. The aim of this scoping review was to identify PROs and PROMs applied for this population.

**Methods:**

The review followed the Joanna Briggs Institute guidelines. Five databases were searched: Medline (OVID), Scopus, Web of Science, CINAHL, and PsycINFO (OVID). Data extracted included the PROs assessed, PROMs used, indications for surveillance colonoscopy, and assessment timepoints.

**Results:**

8684 studies were screened, and 91 were included. Eighteen PROs and 12 PROMs were identified. Abdominal discomfort (60%), abdominal pain (60%) and nausea (56%) were the most frequently collected PROs. PROs were predominantly assessed after bowel preparation/before colonoscopy (55%) and 1–2 days after colonoscopy (48%). Hospital Anxiety and Depression Scale (33.3%), Short Form-36 (33%), the European Organization for Research and Treatment of Cancer Quality of Life Questionnaire 30-item (27%), and EQ-5D-5L (20%) were the most frequently used PROMs.

**Conclusion:**

There is variability in PROs and PROMs applied. This highlights the need for consensus on a standardised set of PROs to be assessed and PROMs to facilitate consistent and reliable data collection to better inform implementation and improve healthcare quality.

**Supplementary Information:**

The online version contains supplementary material available at 10.1007/s11136-026-04270-4.

## Abstract (plain language summary)

The assessment of patient-reported outcomes is recommended as a key indicator of the quality of healthcare service delivery. Individuals at elevated risk for colorectal cancer need frequent colonoscopies and regular encounters with the health care system. Symptoms such as abdominal pain and vomiting are commonly reported in association with bowel preparation for colonoscopy, as well as complications following the procedure. It is therefore important to assess the patient-reported outcomes associated with colonoscopy in this population. However, there is a paucity of evidence on the standardised set of patient-reported outcomes to be assessed and patient-reported outcome measures to be applied. Our study identified the most frequently assessed patient-reported outcomes and the most frequently used measures. Notably, there was considerable variability in the patient-reported outcomes assessed and measures used, the timing of their assessment in relation to the colonoscopy procedure, as well as across different indications for surveillance colonoscopy. This shows the need to standardise patient-reported outcomes and measures to be applied in this population and at what timepoints. This will facilitate consistent and reliable data collection to better inform implementation and improve healthcare quality.

**Supplementary Information:**

The online version contains supplementary material available at 10.1007/s11136-026-04270-4.

## Introduction

Colorectal cancer (CRC) is the fourth most prevalent cancer and the second leading cause of cancer-related mortality globally [1]. The incidence of CRC and associated mortality can be reduced through colonoscopy, which enables early detection and removal of precancerous polyps [2]. Regular colonoscopy for CRC surveillance is recommended in individuals at elevated risk of CRC, such as a personal history of colorectal neoplasia, genetic high-risk conditions to CRC, family history of CRC and having inflammatory bowel diseases (IBD) [3]. However, the colonoscopy procedure is invasive and associated with negative impacts on patient-reported outcomes (PRO). These include effects of the bowel preparation for colonoscopy, such as nausea, bloating or pain, and complications from the procedure such as bleeding and perforation [4].

Assessing individuals’ PROs, a report of the patient’s perception of their health condition obtained directly from the patient such as symptoms [5], is essential for identifying patient needs, enhancing the quality of care, and guiding healthcare providers in improving service delivery [6]. PROs include reports on quality of life or health-related quality of life (HRQoL), functional status and symptoms such as nausea, pain, and anxiety [7, 8].

PROs are especially important for individuals undergoing regular colonoscopy surveillance, as they frequently interact with the healthcare system. Routine collection of PROs can optimize clinical care standards and encourage greater participation in surveillance programs and ultimately contribute to a reduction in CRC incidence. Validated tools designed to assess PROs are known as patient-reported outcome measures (PROMs). Validated PROMs are important for systematically capturing individual PROs in a standardized manner that facilitates effective evaluation of a given intervention [9]. The International Consortium for Health Outcomes Measurement has developed standard sets of PROs and recommendations for PROMs in various conditions including cancer, however, there is no core set of PROs to be assessed in a population undergoing regular cancer surveillance [10].

Although the existing literature provides insights into PROs and PROMs in populations with CRC [11], there is a paucity of such evidence in individuals undergoing regular colonoscopy surveillance. There is no consensus and guidance on PROMs that are fit for purpose in this population, and what the appropriate timing of PRO assessment should be in relation to the colonoscopy procedure (e.g. before or after the procedure). In addition, it is not clear if different PROs should be assessed in relation to different risk factors and indications for surveillance colonoscopy such as a personal history and genetic high-risk conditions for CRC.

This scoping review was undertaken to map existing evidence, identify gaps in measurement and reporting of outcomes for individuals undergoing colonoscopy surveillance and inform future research and clinical practice. Therefore, the aim was to identify PROs and PROMs that have been applied in individuals undergoing regular colonoscopy for CRC surveillance, assessment timepoints in relation to the colonoscopy procedure, and if these differed based on the patient risk factors or indication for surveillance colonoscopy.

## Methods

### Study design

This scoping review was conducted according to the Joanna Briggs Institute (JBI) guidelines [12]. A scoping review was chosen, as it is preferable to map existing studies to answer broader questions, and to address knowledge gaps [13]. The study protocol was registered with the International Platform of Registered Systematic Review and Meta-analysis Protocols (INPLASY); registration number: INPLASY202480008.

## Inclusion and exclusion criteria

*Inclusion criteria*: Empirical studies assessing PROs in individuals at elevated risk for CRC such as a personal or family history of CRC or polyps and genetic high-risk conditions for CRC such as Lynch syndrome undergoing colonoscopy surveillance were included. The review included experimental studies (randomized and non-randomized), analytical observational studies (prospective and retrospective cohort studies), case–control studies and cross-sectional studies (descriptive and analytical). We included only studies published in English, as our team did not include reviewers proficient in other languages.

*Exclusion criteria*: Studies conducted among patients with inflammatory bowel disease (IBD) were excluded, as these individuals have regular colonoscopy to monitor the effectiveness of treatment or screen for complications and not for surveillance. Commentaries, editorial letters, reviews, conference proceedings and case reports were also excluded.

## Databases searched

The databases included MEDLINE (Ovid), Scopus, Web of Science, CINAHL, and PsycInfo (Ovid). The initial search was conducted from database inception to July 26, 2024, and was subsequently updated to March 23, 2026.

## Search strategy

The search strategy was prepared using a combination of Medical Subject Headings (MeSH) terms and keywords related to the study question. The full search strategy is provided in the supplementary information (SI 1). General search terms with reference to the target population (elevated risk for CRC), PROs and PROMs, and clinical context as undergoing regular colonoscopy surveillance were also used.

## Source of evidence selection

Results from the databases were imported into the reference manager software EndNote 21 to remove duplicates (Clarivate Analytics, version 21.2.0.17387, January 2022, USA), and then imported into the systematic review software, Covidence (Veritas Health Innovation, Melbourne, Australia). Title/abstract screening and full-text review were performed by two independent reviewers, with a third reviewer to resolve any disagreements.

## Data extraction

Data were extracted by two independent reviewers with disagreements resolved through discussions or with a third reviewer. The extracted data included details of the study (country, study design, study setting, study period, number of study participants, sex, age), indication for colonoscopy, PROs and PROMs used and timing of PROs assessment in relation to colonoscopy procedure (SI 2).

## Data analysis

Descriptive statistics were generated (using Microsoft Excel). These included the frequency and percentage of identified PROs and PROMs, relative to time point at which they were assessed and the indication for colonoscopy.

## Results

### Literature search

**Fig. 1:**
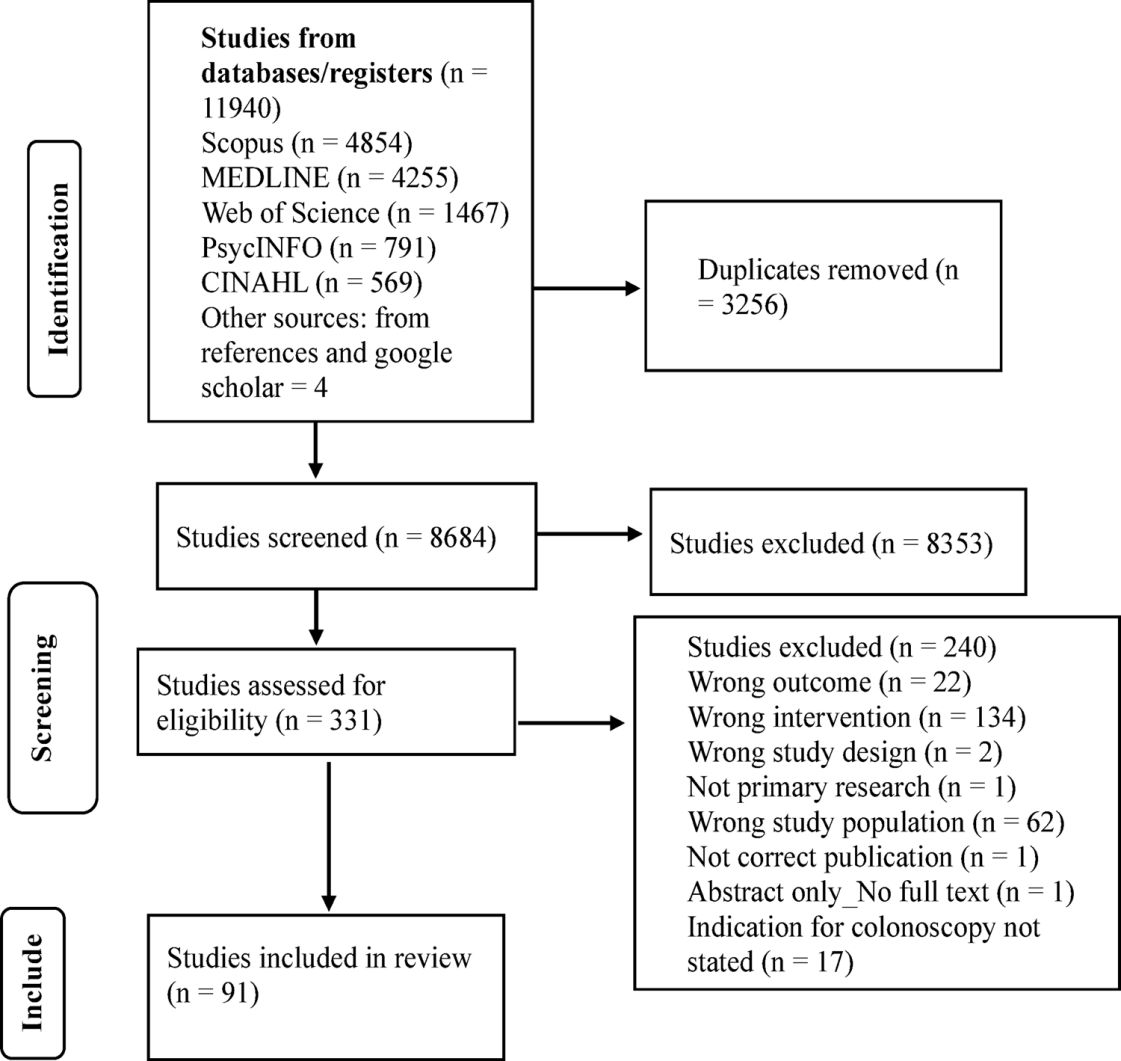
PRISMA Flow diagram for the scoping review process

A total of 11,940 studies were identified, with 3256 duplicates removed. The remaining 8,684 studies were screened; 331 studies were retrieved for full-text review, and 91 met the inclusion criteria (Fig. 1).

## Socio-demographic and clinical related information of the studies

Studies included in the review were undertaken in 25 countries, mostly from the USA (n = 14, 15%), followed by Italy (n = 11, 12%) and China (n = 10, 11%) (SI 3). Most studies were randomized controlled trials (RCTs) (n = 58, 64.0%), and cross-sectional studies (n = 22, 24%). The details of each study and citations are provided in the supplementary information (SI 4).

## Patient-reported outcomes (PROs)

**Fig. 2:**
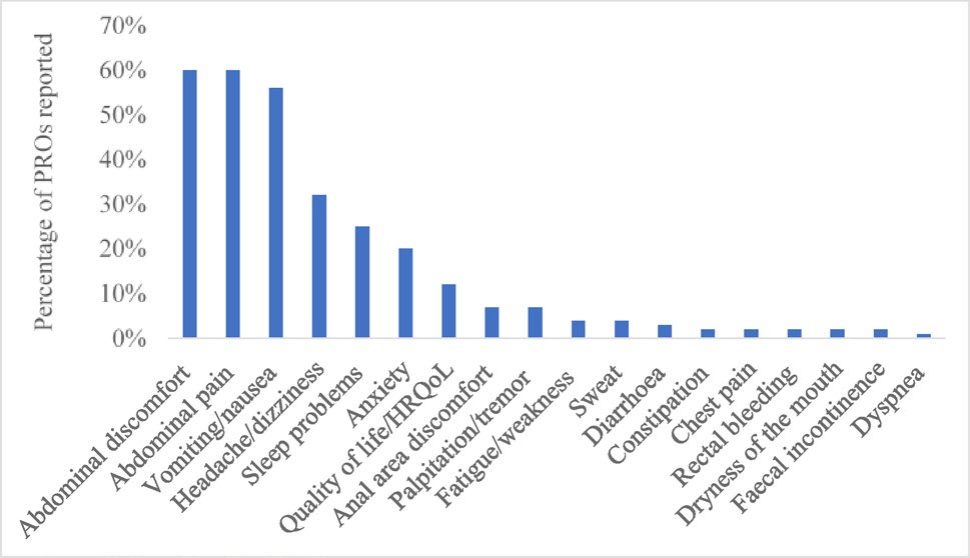
Percentage (%) of PROs used in individuals undergoing colonoscopy surveillance (n = 91). *Footnote*: HRQoL: Health related quality of life. *NB*: Percentage is calculated based on how many times each PRO was included across the 91 studies

Eighteen PROs were identified, and the most frequently measured PROs were abdominal discomfort (60%) [14–68], abdominal pain (60%) [15, 16, 18–22, 24–26, 28–30, 32–35, 37, 39, 41–49, 51, 53, 54, 59–63, 66, 68–85], nausea/vomiting (56%) [14–16, 18, 19, 21, 22, 24–28, 30–35, 37, 38, 41–43, 45–48, 50, 51, 54, 56, 59–67, 70, 72, 73, 76, 77, 79, 82, 83, 86–88], and headache/dizziness (32%) [14, 15, 19, 24–28, 31, 36, 38, 41, 42, 46, 48, 50, 54, 62, 64, 65, 70, 72, 77, 86, 88–90]. Sleep problems (24%) [73, 82, 87], anxiety (20%) [40, 49, 52, 55, 75, 78, 80; 90–100], and HRQoL (13%) [40, 75, 81, 88–90, 92, 101–103] were also highly reported (Fig. 2).

## Patient-reported outcome measures (PROMs)

**Fig. 3:**
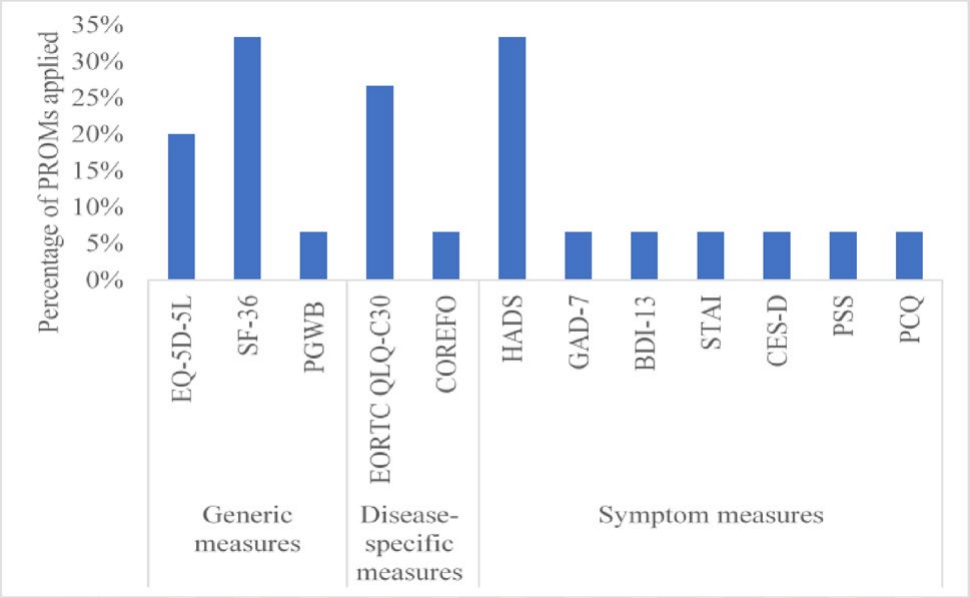
PROMs used in individuals at elevated risk for CRC undergoing colonoscopy surveillance (n = 15). *Footnote*: SF-36: Short-Form 36; PGWB: Psychological General Well-Being; EORTC QLQ-C30: European Organization for Research and Treatment of Cancer Quality of Life Core Questionnaire; CORFEO: Colorectal Functional Outcome; HADs: Hospital Anxiety and Depression scale; GAD-7: General Anxiety Disorder; BDI-13: Beck Depression Inventory-13 item; STAI: State-Trait Anxiety Inventory; CES-D: Centre for Epidemiological Studies Depression Scale; PSS: Perceived Stress Scale; PCQ: Psychological Consequences Questionnaire. *NB*: Percentage is calculated based on how many times each PROM was included across the 15 studies that used PROMs

Twelve validated PROMs were applied across 15 studies [40, 75, 78, 81, 88–94, 96, 97, 101, 103], mostly assessing HRQoL (42%). The most used generic HRQoL measures were the Short Form -36 (SF-36) [40, 75, 88, 93, 103] and EQ-5D-5L [90, 92, 101] in 33% and 20% of studies while the European Organization for Research and Treatment of Cancer Quality of Life Core Questionnaire (EORTC QLQ-C30) [88–90, 101] was the most used cancer specific PROM (27%). The most used symptom scale was the Hospital Anxiety and Depression scale [75, 78, 90, 93, 96] applied in 33% of studies. All other PROMs used [81, 91, 92, 94, 97, 103] are showed in Fig. 3.

## Time points at which PROs were assessed and PROMs used

**Fig. 4:**
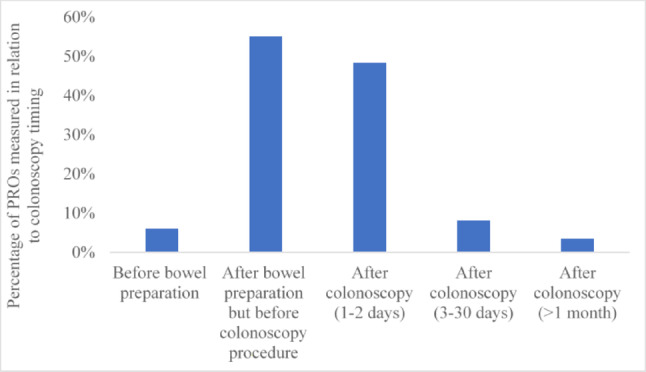
PROs assessment time point in relation to the colonoscopy procedure (n = 91). *NB*: PROs were assessed at more than one time point in 19 studies. As a result, these studies contributed data to more than one time point

**Fig. 5:**
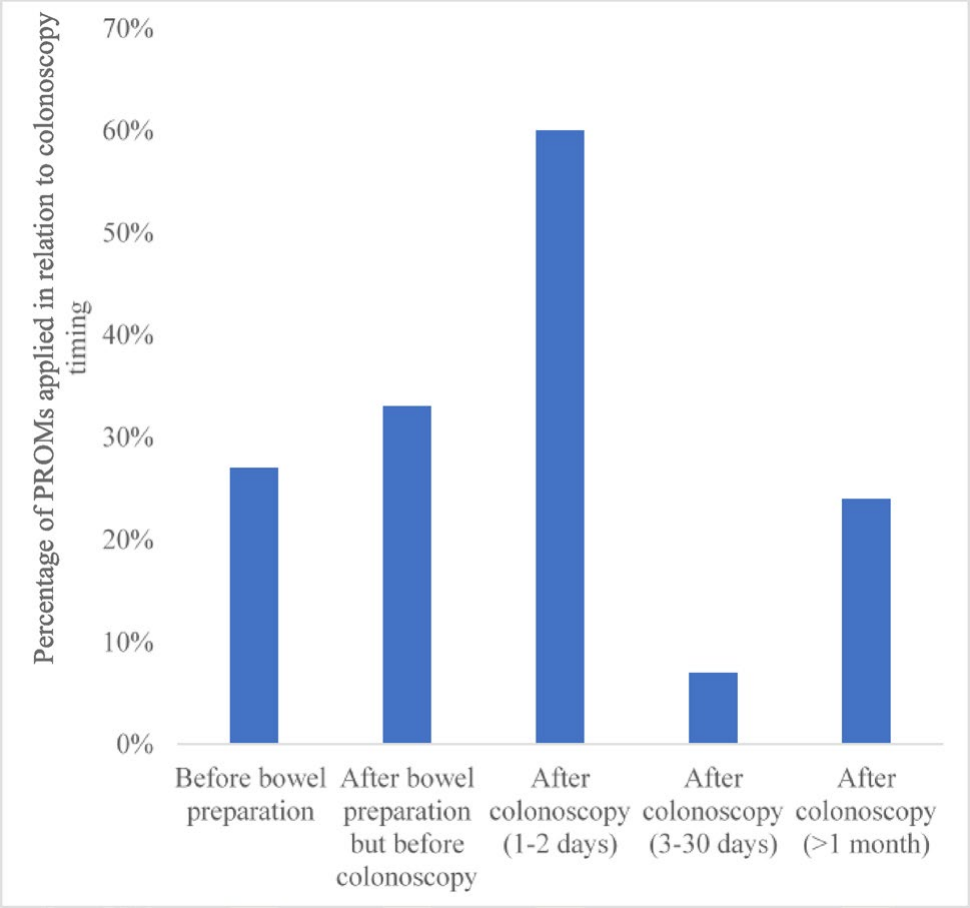
Time points of PROMs in relation to colonoscopy procedure (n = 15). *NB*: Among the studies that used PROMs, 7 studies have more than one time point. As a result, these studies contributed data to more than one time point

Most studies (55%) assessed PROs after bowel preparation but before colonoscopy, followed by 1–2 days post-colonoscopy (48%) (Fig. 4). Among 15 studies that applied PROMs, the majority (60%) used them 1–2 days after colonoscopy followed by after bowel preparation but before the colonoscopy (33%) (Fig. 5).

## PROs and PROMs based on indication for surveillance colonoscopy

**Fig. 6:**
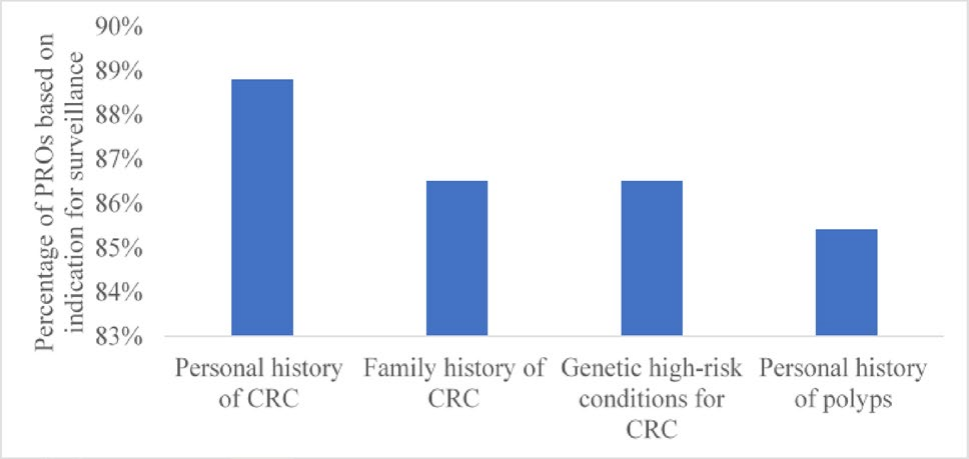
PROs based on indications for surveillance colonoscopy (n = 91). *Footnote*: CRC: Colorectal cancer. *NB*: 76 studies have more than one colonoscopy indications. As a result, these studies contributed data to more than one time

**Fig. 7:**
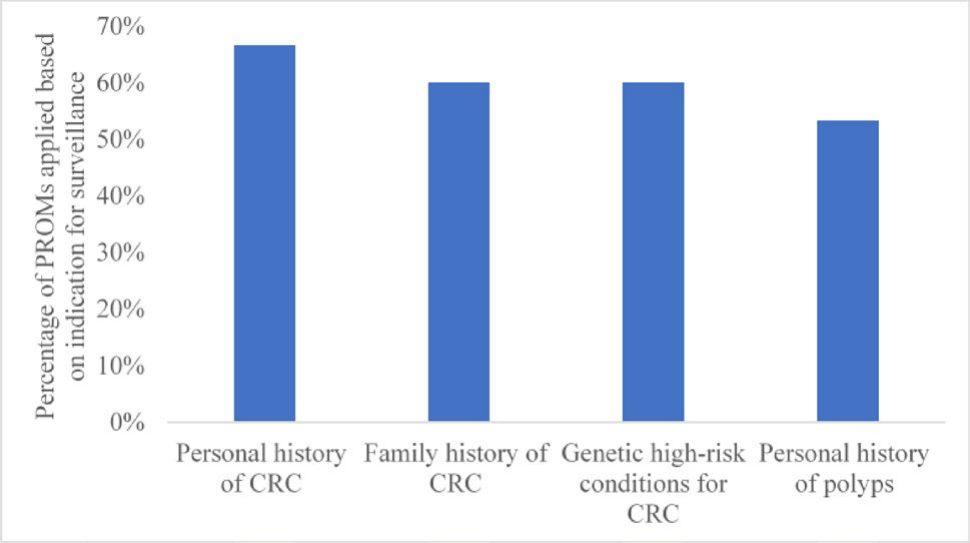
PROMs based on indications for surveillance colonoscopy (n = 15). *Footnote*: CRC: Colorectal cancer. *NB*: 8 studies have more than one colonoscopy indications. As a result, these studies contributed data to more than one time

Most of the studies (89%) assessed PROs in individuals undergoing surveillance colonoscopy due to personal history of CRC followed by family history of CRC (87%) and genetic high-risk conditions for CRC (87%) (Fig. 6). A similar trend was observed in the 15 studies that applied PROMs, 67%, 60% and 60% for individuals undergoing colonoscopy due to a personal history of CRC, a family history of CRC and genetic high-risk conditions, respectively (Fig. 7).

## Discussion

This is the first scoping review to identify PROs assessed and PROMs applied in individuals undergoing regular colonoscopy for CRC surveillance. We identified the most frequently assessed PROs and used PROMs, however, there was no consistency in the PROs and PROMs across studies, and in the timing of assessment.

The most assessed PROs were abdominal discomfort, abdominal pain, vomiting and headache. Findings of this review are consistent with previous reviews on adverse events following colonoscopy [104–106], which identified abdominal discomfort and abdominal pain as the most frequently measured PROs. In addition, a previous systematic review and meta-ethnography on patient reported experiences following colonoscopy, identified discomfort (unspecified discomfort) as a predominant concern [107]. Gastrointestinal symptoms may be attributed to bowel preparation agents, such as polyethylene glycol solutions, which cause abdominal discomfort due to their unpleasant smell and taste [108]. Abdominal pain is a result of the physiological mechanisms during the colonoscopy procedure, particularly bowel distention and ligament stretching to achieve successful caecal intubation [109]. Vomiting and headache could be linked to serum electrolyte disturbance during bowel preparation [110].

The most commonly used PROMs in this population were the HAD, SF-36, EORTC QLQ-C30, and EQ-5D-5L. Individuals undergoing regular surveillance colonoscopy do not typically have symptoms of active cancer such as rectal bleeding or changes in bowel habits. As such, cancer-specific PROMs like the EORTC QLQ-C30 which address functional status, or symptoms related to malignancy, may not be appropriate, yet the generic PROMs do not address their unique concerns including the fear of cancer [111]. The identified PROMs primarily address general health and active cancer: the SF-36 and EQ-5D-5L focus on physical and social functioning, while the HADS assesses general psychological distress, and the EORTC QLQ-C30 measures the functional and symptomatic impact of active cancer. Consequently, a distinct 'domain gap' exists, as these tools fail to capture the unique psychological concerns of this population, such as the specific fear of cancer. Therefore, the selection of PROMs should be guided by their conceptual relevance to surveillance populations, with preference given to measures that integrate physical symptoms associated with the colonoscopy procedure such as abdominal discomfort and anxiety, as these dimensions influence adherence to surveillance recommendations and patient engagement [4, 112].

This review found that there is no consistent timepoint at which PROs are assessed or PROMs applied in relation to the colonoscopy procedure. Additionally, we observed that no specific PROs or PROMs were applied based on the indication for surveillance colonoscopy. Assessment of health outcomes at different timepoints could provide a detailed understanding of the pre-procedure and post-procedure concerns associated with colonoscopy surveillance. In addition, it could provide a better understanding of the immediate and longer-term impacts of colonoscopy surveillance for populations at increased risk of CRC. Together, these findings underscore the importance of standardising outcome measures within surveillance populations according to both assessment timing and indications for surveillance.

## Which PROs and PROMs should be applied in surveillance populations

Integrating the identified PROs such as abdominal discomfort, abdominal pain, vomiting, headache, sleep problems, anxiety and HRQoL, into routine clinical assessments will establish a holistic, patient-centered care model to improve the overall patient outcomes in surveillance. Although this review identified the most used PROMs, no validated, standardized PROM that comprehensively captures all PROs identified as relevant for individuals undergoing regular colonoscopy surveillance. Because HRQoL is multi-dimensional concept, its assessment may incorporate some of the individual PROs highlighted above like anxiety. However, a PROM for use in this population would require assessment of specific adverse events related to the bowel preparation and colonoscopy procedure like abdominal discomfort as well as the fear of cancer and the overall patient health conditions.

## The need for standardisation of PROs and PROMs in surveillance populations

Considerable variability was observed in both the PROs assessed and the PROMs used across the studies, indicating a lack of standardised outcome measurement in this population. Clinically, this limits the interpretability and comparability of findings across different studies. Standardisation of PROs and PROMs would enable more reliable comparisons across studies and surveillance programs, thereby strengthening the evidence base for the development of surveillance guidelines [113].

In practice, a standardised approach would allow consistent monitoring of patient outcomes and systematic incorporation of patient perspectives into care [114]. It would also facilitate longitudinal assessment, enabling clinicians to identify trends and optimise follow-up strategies within surveillance programs. This review highlights the need to establish a core set of PROs, and development of a comprehensive PROM to capture these outcomes that would facilitate consistent evaluation of colonoscopy surveillance services and enhance the integration of patient-reported data into routine clinical practice.

As a limitation, the search was restricted to studies published in English, so relevant studies published in other languages may have been missed. Also 74% of the studies included a mixed population of individuals undergoing colonoscopy surveillance as well as those underdoing colonoscopy due to abdominal symptoms or screening colonoscopy. As such the findings may not only represent the PROs and applied PROMs for individuals specifically undergoing regular colonoscopy surveillance. However, the study included multiple databases and a comprehensive search strategy, and overall, there is a paucity of studies specifically focused only on individuals undergoing colonoscopy for surveillance.

## Conclusion

The most frequently assessed PROs were abdominal discomfort, abdominal pain, vomiting and headache. The most frequently used PROMs were the HAD, SF-36, EORTC QLQ C-30, and EQ-5D-5L. Considerable variability in the PROs assessed and PROMs applied was observed, which limits comparability across surveillance programs and evaluation to inform clinical practice and quality of care. Additionally, there was variability in the time point for PRO assessment and PROMs used in relation to the colonoscopy procedure, and no PRO or PROM specifically tailored to the different indications for surveillance colonoscopy. These findings emphasise the need to establish a core set of PRO and the most appropriate PROMs so as to standardise PRO assessment and enhance the quality of regular colonoscopy surveillance programs for CRC prevention.

## Supplementary Information

Below is the link to the electronic supplementary material.

Supplementary Material 1

## Data Availability

No datasets were generated or analysed during the current study.

## Abbreviations

CRC: Colorectal cancer
EORTC QLQ-C30: The European Organization for Research and Treatment of Cancer Quality of Life Core Questionnaire
HADs: Hospital anxiety and depression scale
HRQoL: Health related quality of life
INPLASY: International platform of registered systematic review and meta-analysis protocols
PROMs: Patient-reported outcome measures
PROs: Patient-reported outcomes
RCT: Randomized controlled trial
NRCT: Non-randomized controlled trial
SF-36: Short form-36
