# Supplementary material for: Patient-reported outcomes and measures applied in individuals undergoing colonoscopy surveillance for colorectal cancer: a scoping review
Source: Qual Life Res. 2026 Jun 6;35(7):170. doi: 10.1007/s11136-026-04270-4 (PMC13242399; doi:10.1007/s11136-026-04270-4)
Supplement: Supplementary file 1 — Supplementary Material 1 [file 11136_2026_4270_MOESM1_ESM.docx]

**Supplementary information (SI)**

**Title**: Patient-reported outcomes and measures applied in individuals undergoing colonoscopy surveillance for colorectal cancer: A scoping review

**Authors:** Wudneh Simegn Belay^1,2^, Erin L. Symonds^1,3^, Melkalem Mamuye Azanaw^1,4^, Meseret Derbew Molla^1,5^, Molla M. Wassie^1^, Geraldine Laven-Law^6,7^, Martha A. Menberu^1^, Muktar Ahmed^1^, Billingsley Kaambwa^1^, Norma B. Bulamu^1^

^1^Flinders University, Flinders Health and Medical Research Institute, College of Medicine and Public Health, Bedford Park, South Australia, Australia.

^2^Department of Social and Administrative Pharmacy, School of Pharmacy, College of Medicine and Health Sciences, University of Gondar, Gondar, Ethiopia.

^3^Gastroenterology Department, Flinders Medical Centre, Southern Adelaide Local Health Network, Bedford Park, South Australia, Australia.

^4^Department of Public Health, College of Health Sciences, Debre Tabor University, Debre Tabor, Ethiopia.

^5^Department of Biochemistry, School of Medicine, College of Medicine and Health Sciences, University of Gondar, Gondar, Ethiopia

^6^South Australian Immunogenomics Cancer Institute, Adelaide University, Adelaide, South Australia, Australia

^7^College of Health, Adelaide University, Adelaide, South Australia, Australia

**Corresponding author:** [**sime0034@flinders.edu.au**](mailto:sime0034@flinders.edu.au)

**SI 1: Search strategy**

The following search strategy was developed for MEDLINE (Ovid) and adapted for use in other databases.

(((cancer* OR tumo?r* OR Neoplas* OR adenocarcinoma*) adj4 (colorectal OR colon* OR rectal OR rectum* OR bowel OR "large intestine*")) OR ((cecal OR famil* OR colorect* OR colon* OR rect* OR intestin* OR bowel*) adj3 (cancer* OR neoplas* OR tumo?r OR carcinoma OR Adenoma* OR adenocarcinoma OR malignan* OR polyp* OR Serrated)) OR "Juvenile polyposis syndrome" OR "Lynch syndrome" OR "mutyh associated polyposis" OR "peultz jegher syndrom" OR "Serrated polyposis syndrome" OR "Juvenile polyp" OR "traditional serrated adenoma" OR "Sessile serrated polyp" OR Acromegaly OR "Crohn's colitis" OR "Ulcerative colitis") AND ((Population adj3 surveillance) OR Colonoscop* OR "elevated risk" OR "high?risk" OR "above average risk" OR "personal history" OR "family history" OR "follow?up" OR "post?polypectomy" OR polypectomy OR (polyp adj3 removal) OR "polyp resection" OR "early detection of cancer") AND ("patient* reported outcome*" OR "Health related quality of life" OR HRQoL OR HRQL OR HRQOL OR QOL OR OQOL OR "quality of life" OR (fear adj4 cancer) OR pain* OR mobility OR "self?care" OR discomfort OR anxi* OR stress* OR emotion* OR "sleep* disorder*" OR (appetite adj4 loss) OR dyspnoea OR dyspnea OR diarrhoea OR constipate* OR depress* OR vomit* OR "psychological well?being" OR fatigue) OR ("patient reported outcome measure*" OR PROM* OR survey* OR Questionnaire* OR "Self?report" OR "Health stat* indicator*" OR instrument* OR measure* OR indices OR index or inventor* OR tool* OR scor* OR indicator* OR scal* OR rat* OR assess* OR survey* OR "health utility indicator*" OR HUI OR "short?form 6*" OR SF6* OR "short?form 12*" OR "SF 12*" OR "short?form 36*" OR "SF 36*" OR "euro?qol" OR "EQ?5D*" OR "Assessment of Quality of Life measure*" OR AQOL* OR QoLI OR "quality of well? being" OR "quality of wellbeing" OR QWB OR (("quality of wellbeing" OR QWB) adj3 ("15 dimension*" OR "16 dimension*" OR "17 dimension*" OR "15?D" OR "16?D" OR "17?D")) OR "EORTCQLQ?C30" OR "EORTC Core Quality of Life questionnaire*" OR "EORTC QLQ?C30" OR EORTC* OR FACT?G OR "Functional Assessment of Cancer Therapy General" OR FACT* OR "Outcome Assessment" OR "patient acceptance of health care"))

**SI 2: Data extraction format**

| **Sn** | **Items** | **Answers/Possible options** |
| --- | --- | --- |
| Part 1: Socio demographic and clinical characteristics | | |
|  | Author and year |  |
|  | Title |  |
|  | Country |  |
|  | Study design |  |
|  | Centre of study (single, multiple) |  |
|  | Study period |  |
|  | Number of participants |  |
|  | Gender |  |
|  | Mean/median age |  |
|  | Study population based on colonoscopy indication | 1. Personal history, 2. Family history, 3. genetic high-risk conditions for CRC (lynch syndrome, familial adenomatous polyposis…or others), 4. History of polyps |
|  | Assessment time in relation to colonoscopy procedure | Before, immediate, after (specific time) |
| Part 2: Patient reported outcomes (PROs) and patient reported outcome measures (PROMs) used | | |
|  | Name of PROs |  |
|  | Name of PROMs |  |
|  | Types of PROMs | If mentioned (generic, cancer specific, symptom specific, diseases specific, others) |

**SI 3: Figure 1**

Figure 1: Distribution of studies by country to identify PROs and measures applied in individuals undergoing colonoscopy surveillance for CRC (n = 91)

**SI 4: Table 1**

**Table 1: Characteristics of studies included in the scoping review (n = 91)**

| **Author/year** | **Country** | **Study design** | **Study setting** | **Study period** | **Number of participants** | **Male** | **Female** | **Participants’ mean/median age with SD** |
| --- | --- | --- | --- | --- | --- | --- | --- | --- |
| Abgrall-Barbry 2012 [1] | France | Cross-sectional | Multi | July-August 2012 | 94 | 35 | 59 | 56.2 ± 9.8 |
| Alhassan 2024 [2] | Saudi Arabia | Cross-sectional | Single | 2022 | 14 | 5 | 9 | 34.93 ± 12.83 |
| Arieira 2021 [3] | Portugal | RCT | Single | January-July 2019 | 477 | 328 | 149 | 61 and 61 |
| Augestad 2013[4] | Norway | RCT | Multi | 2007-2011 | 29 | 65 | 45 | 66.7 and 64.0 |
| Backes 2016 [5] | Netherlands | RCT | Multi | - | - | - |  | - |
| Barkun 2020 [6] | Canada | RCT | Multi | - | 1750 | 799 | 952 | 56.3 and 61.3 |
| Bhandari 2023 [7] | USA | RCT | Multi | July 13, 2020-February 5, 2021 | 500 | 206 | 294 | 56.2 ± 11.7 |
| Brotons 2024 [8] | Spain | cross-sectional | Multi | February 2019-June 2020 | 1753 | 944 | 805 | 61 |
| Bulamu 2024 [9] | Australia | Prospective cohort | Multi | March 2017 and July 2019 | 246 | 134 | 112 | 64 |
| Castro 2019 [10] | USA | RCT | Single | January 2016- August 2017 | 300 | 148 | 152 | 56.7 ± 10.6 and 58.9 ± 10.0 |
| Chan 2014 [11] | Malaysia | Cross-sectional | Single | August 2012- March 2013 | 303 | 144 | 159 | 62.0 ± 14.4 |
| Chan 2011 [12] | Malaysia | Cross- sectional | Single | October 2006- March 2007 | 501 | 256 | 245 | 60.1 ± 14.0 |
| Cheng 2022 [13] | China | RCT | Single | December 2017- February 2018 | 318 | 164 | 154 | 51.1 ± 13.0 |
| Chen 2024 [14] | China | RCT | Multi | June 2020 - March 2021 | 900 | 489 | 411 | 49.4 ± 11.6 and 49.7 ± 12.4 |
| Chen 2018 [15] | Taiwan | RCT | Single | May 2016- September 2016 | 120 | 56 | 64 | 52.9 ± 12.7 and 54.5 ± 11.1 |
| Choi 2014 [16] | Korea | RCT | Multi | June 2013-November 2013 | 107 | 57 | 50 | 57.0 ± 12.5 and  57.2 ± 13.6 |
| Daniels 2012 [17] | USA | Cross -sectional | Multi | 2008-2009 | 412 | 233 | 179 | 56 ± 11.68 |
| Denis 2004 [18] | France | Prospective | Single | June 1 - October 31, 2002 | 500 | 254 | 246 | - |
| Eckardt 2008 [19] | USA | RCT | Single | - | 368 | 209 | 159 | 56.7 ± 9.5 and 57.1 ± 8.9 |
| Fernández-Landa 2019 [20] | Spain | cross-sectional study | Multi | November-December 2015 | 432 | 219 | 213 | 59.8 ± 0.4 |
| Ge 2023 [21] | China | RCT | Multi | January 2017- December 2019 | 1155 | 881 | 274 | 72.4 ± 8.4, and 71.5 ± 9.2 |
| Gerard 2012 [22] | USA | RCT | Single | February 2010- February 2011 | 600 | 329 | 271 | 62.8 ± 12.9 |
| Hamada 2023 [23] | Japan | RCT | Single | October 2013- November 2017 | 219 | 0 | 218 | 62.8 ± 12.9 |
| Hori 2024 [24] | Japan | single arm non-randomized | Multi | July- November 2023 | 137 | 60 | 77 | 56 |
| Jensch 2010 [25] | Netherlands | Cross-sectional | Single | - | 173 | 107 | 66 | - |
| Jung 2022 [26] | Korea | RCT | Single | - | 347 | 175 | 172 | 69.3 ± 5.6 and 69.3 ± 5.0 |
| Jung 2016 [27] | Republic of Korea | RCT | Single | - | 222 | 113 | 109 | 55 ± 12.0 and 53 ± 13.0 |
| Jun 2017 [28] | Republic of Korea | RCT | Single | August 2014 - November 2015 | 204 | 118 | 86 | 54.6 ± 11.5 and 54.3 ± 9.4 |
| Katz 2013 [29] | USA | RCT | Multi | - | 598 | 217 | 381 | 56.8 and 56.2 |
| Keane 2020 [30] | New Zealand | Cross-sectional | Single | 2008–2015 | 434 | 268 | 166 |  |
| Kim 2017 [31] | Korea | RCT | Multi | November 2015- March 2016 | 167 | 94 | 73 | 57.1 ± 10.0 and  55.7 ± 12.2 |
| Kim 2014 [32] | Korea | RCT | Single | July- October 2013 | 200 |  |  | 56 ± 12 |
| Kim 2020 [33] | Korea | RCT | Single | August 2017- January 2018 | 168 | 73 | 95 | 48.14 ± 12.20 and 52.88 ± 11.19 |
| Kim 2014 [34] | Republic of Korea | RCT | Single | - | 184 | 85 | 99 | 52.8 ± 10.4 and 54.0 ± 9.5 |
| Leung 2009 [35] | USA | RCT | Single | May 9, 2008- July 28, 2008 | 56 | 51 | 5 | 60 ± 6.6 and 59 ± 8.6 |
| Liljegren 2004 [36] | Sweden | Cross sectional | Single | - | 240 | 96 | 144 | 48 |
| Lynch 2015 [37] | USA | RCT | Single | - | 191 | 177 | 14 | 62.8 and 62.3 |
| Mancini 2021[38] | Italy | Cross- sectional | Single | January 1,2015- June 30, 2016 | 62 | 31 | 31 | 60.8 ± 9.4 |
| Manes 2013 [39] | Italy | RCT | Multi | January- June 2011 | 285 | 161 | 24 | 60.9 ± 12.3 and 57.8 ± 14.4 |
| Manes 2014 [40] | Italy | RCT | Multi | January 2012- June 2012 | 862 | 432 | 430 | 58.5 ± 14.4 and 59.8 ± 14.5 |
| Nalankilli 2021 [41] | Australia | RCT | Single | - | 164 | 95 | 69 | 56.7 ± 13.9 and 57.3 ± 17.5 |
| Nicholson 2005 [42] | Australia | Cross-sectional | Single | October 1, 2002-March 31, 2003 | 447 | 200 | 247 | - |
| Niv 2012 [43] | Israel | Prospective cohort | Single | - | 100 | 56 | 44 | 61.8 ± 13.9 |
| O'Leary 2011 [44] | USA | Cross -sectional | Single | 2008- 2009 | 93 | 43 | 47 | 56.63 ± 11.5 |
| Parente 2014 [45] | Italy | Prospective | Multi | February-June 2012 | 599 | 308 | 291 | 56.7 |
| Pohl 2015 [46] | Germany | RCT | Multi | - | 398 | 213 | 185 | 59.8 ± 9.2 |
| Pontone 2011 [47] | Italy | RCT | Single | May 2009-October 2010 | 144 | 76 | 68 | 59.5 |
| Pontone 2022 [48] | Italy | prospective | Single | - | 123 | 59 | 64 | 57.70 ± 15.0 |
| Quintana 2023 [49] | Spain | prospective cohort | Multi | June 2010- December 2012 | 2448 | 1557 | 891 | 68.36 ± 11.01 |
| Regev 1998 [50] | Israel | RCT | Single | - | 68 | 32 | 36 | 60 ± 15 and 62 ± 14 |
| Repici 2021 [51] | Italy | RCT | Multi | January 2019- October 2019 | 388 | 184 | 204 | 59.5 ± 11.3 and 60.1 ± 12.0 |
| Rex 2014 [52] | USA | RCT | Multi | August 25, 2008- November 21, 2008 | 371 | 178 | 193 | 56.9 ± 11.4 and 56.8 ± 11.0 |
| Ristvedt 2003 [53] | USA | Prospective cohort | Single | July 2000-May 2001 | 120 | 53 | 67 | - |
| Rivas 2014 [54] | USA | RCT | Single | October 2009-November 2012 | 206 | 126 | 80 | 56.7 |
| Rosati 2016 [55] | Italy | RCT | Multi | 1998 - 2006 | 1228 | 746 | 482 | 63.5 and 64.3 |
| Sadeghi 2022 [56] | Iran | RCT | Single | February 2021 - March 2022 | 382 | 172 | 210 | 49.47 ± 11.12 and 48.88 ± 11.75 |
| Samarasena 2022 [57] | USA | RCT | Multi | October 2014 - October 2017 | 195 | 132 | 63 | 57.9 and 59.7 |
| Scaglione 2023 [58] | Italy | RCT | Multi | - | 289 | 151 | 138 | 60.4 ± 12.9 |
| Seo 2013 [59] | Korea | RCT | Single | January-April 2012 | 197 | 101 | 96 | 54.6 |
| Shafer 2018 [60] | Canada | Cross-sectional | Multi | August 2015-June 2016 | 1296 | 616 | 680 | 56 |
| Shavakhi 2021 [61] | Iran | RCT | Single | September 2018-September 2019 | 246 | 106 | 140 | 50.5 ± 12.1 and 49.2 ± 13.6 |
| Sheng 2021 [62] | China | RCT | Single | November-December 2019 | 180 | 84 | 96 | 48.0 ± 22.5 and 44.0 ± 25.0 |
| Sirinawasatien 2022 [63] | Thailand | RCT | Single | December 2019- June 2021 | 140 | 84 | 56 | 58.7 ± 10.8 |
| Suthadsanavijit 2022 [64] | Thailand | RCT | Single | - | 66 | 30 | 36 | 58.85 ± 13.93 and 58.13 ± 13.97 |
| Tajika 2013 [65] | Japan | RCT | Single | - | 253 | 144 | 108 | 65.3 ± 9.9 and 66.3 ± 9.6 |
| Tarr 2014[66] | New Zealand | Cross-sectional | Single | 1996- 2012 | 148 | 56 | 92 | 57.9 |
| Taylor 2010 [67] | United Kingdom | Prospective | Single | April 2007- April 2009 | 56 | 26 | 30 | 64 |
| VanBokhorst 2025 [68] | Netherlands | Cross-sectional | Multi | July 2023- February 2024 | 243 | 118 | 125 | 65 (58-71) |
| VanKeulen 2018 [69] | Netherlands | cross-sectional | Multi | December 1,2016-May 31, 2017 | 47 | 20 | 27 | 61 |
| VanLiere 2023 [70] | Netherlands | cross-sectional | Multi | November 2021- March 2022 | 197 | 91 | 106 | - |
| Van2012 [71] | Netherlands | RCT | Single | - | 116 | 58 | 58 | 50 ± 30 |
| Vassallo 2024 [72] | Italy | RCT | Multi | March- September 2022 | 446 | 221 | 225 | 59.3 ± 14.9 |
| Voiosu 2014 [73] | Romania | cross-sectional | Multi | - | 452 | 215 | 239 | 56.3 ± 15 |
| VonAtzingen 2014 [74] | Brazil | Prospective cohort | Single | September 2010- May 2012 | 85 | 32 | 53 |  |
| Walker 2022 [75] | USA | RCT | Single | - | 213 | 86 | 127 | 57.6 ± 0.8 |
| Walter 2021 [76] | Germany | RCT | Multi | November 2017-January 2019 | 489 | 261 | 228 | 56.8 ± 9.3 and 55.0 ± 7.6 |
| Walter 2019 [77] | Germany | RCT | Multi | December 2015-January 2017 | 495 | 242 | 253 | 47.2 ± 14.8 and 47.5 ± 13.6 |
| Wang 2019 [78] | China | RCT | Single | - | 384 | 217 | 167 | 51.5 ± 12.1 |
| Wang 2026 [79] | China | RCT | Single | June 2024-June 2025 | 200 | 100 | 100 | 53.5 (IQR 46–62) |
| Wattchow 2006 [80] | Australia | RCT | Multi | March 1998- March 2001 | 203 | 117 | 86 | - |
| Wen 2017 [81] | Taiwan | RCT | Single | January 2016- December 2016 | 153 | 48 | 105 | 49.9 ± 14.2 |
| Williams 2006 [82] | UK | Cross-sectional | Single | - | 45 | - |  | 44 |
| Xiong 2023 [83] | China | Retros  pective | Single | June 2020-December 2021 | 1251 | 674 | 577 | 56.12 ± 13.06 and  56.73 ± 12.26 |
| Yang 2020 [84] | Republic of Korea | RCT | Multi | April 10- July 12, 2018 | 228 | 83 | 145 | 46.4 ± 11.2 and 48.2 ± 11.3 |
| Yang 2017 [85] | Republic of Korea | RCT | Multi | November 2015- April 2016 | 199 | 116 | 83 | 51.2 ± 9.3 and 53.4 ± 8.5 |
| Zhang 2023 [86] | China | RCT | Multi | January 6, 2022- April 30, 2022 | 548 | 296 | 252 | 46.36 ± 11.53 and 45.08 ± 11.82 |
| Zhang 2018 [87] | China | RCT | Multi | April 15- July 15, 2015 | 579 | 338 | 241 | 45.5 ± 14.7 and 44.7 ± 12.8 |
| Marmo 2010 [88] | Italy | RCT | Single | - | 868 | 496 | 372 | 58.3 ± 14.8 |
| Fang 2017 [89] | China | RCT | Single | - | 300 | 190 | 110 | 46.8 ± 12.2 and 48.7 ± 11.3 |
| Tarr 2013 [90] | New Zealand | Cross-sectional | Single | February 1996- February 2012 | 148 | 56 | 92 | 57.9 |
| Moloney 2019 [91] | Australia | Cross-sectional | Single | 2008 - 2018 | 138 | 75 | 63 | 67 |

References

1. Abgrall-Barbry, G., et al., *Depressive mood and subsequent cancer diagnosis in patients undergoing a colonoscopy.* Psychosomatics, 2012. **53**(4): p. 356-362.

2. Alhassan, N., et al., *Surveillance compliance and quality of life assessment among surgical patients with familial adenomatous polyposis syndrome.* Journal of Epidemiology and Global Health, 2024. **14**(1): p. 86-93.

3. Arieira, C., et al., *Bowel cleansing efficacy for colonoscopy: prospective, randomized comparative study of same-day dosing with 1-L and 2-L PEG+ ascorbate.* Endoscopy International Open, 2021. **9**(11): p. E1602-E1610.

4. Augestad, K.M., et al., *Cost-effectiveness and quality of life in surgeon versus general practitioner-organised colon cancer surveillance: a randomised controlled trial.* BMJ open, 2013. **3**(4): p. e002391.

5. Backes, Y., et al., *Endoscopic mucosal resection (EMR) versus endoscopic submucosal dissection (ESD) for resection of large distal non-pedunculated colorectal adenomas (MATILDA-trial): rationale and design of a multicenter randomized clinical trial.* BMC gastroenterology, 2016. **16**: p. 1-10.

6. Barkun, A.N., et al., *The Bowel CLEANsing national initiative: a low-volume same-day polyethylene glycol (PEG) preparation vs low-volume split-dose PEG with bisacodyl or high-volume split-dose PEG preparations—a randomized controlled trial.* Official journal of the American College of Gastroenterology| ACG, 2020. **115**(12): p. 2068-2076.

7. Bhandari, R., et al., *Comparison of a novel, flavor-optimized, polyethylene glycol and sulfate bowel preparation with oral sulfate solution in adults undergoing colonoscopy.* Journal of Clinical Gastroenterology, 2023. **57**(9): p. 920-927.

8. Brotons, A., et al., *Colonoscopy Satisfaction and Safety Questionnaire based on patient experience (CSSQP): A valuable quality tool for all colonoscopies.* Gastroenterología y Hepatología, 2024. **47**(1): p. 1-13.

9. Bulamu, N.B., et al., *Health utility assessments in individuals undergoing diagnostic and surveillance colonoscopy: improved discrimination with a cancer-specific scale.* Cancer Causes & Control, 2024. **35**(2): p. 347-357.

10. Castro, F.J., et al., *Randomized controlled trial: split-dose and same-day large volume bowel preparation for afternoon colonoscopy have similar quality of preparation.* Journal of Clinical Gastroenterology, 2019. **53**(10): p. 724-730.

11. Chan, W.-K., et al., *Split-dose vs same-day reduced-volume polyethylene glycol electrolyte lavage solution for morning colonoscopy.* World Journal of Gastroenterology: WJG, 2014. **20**(39): p. 14488.

12. Chan, W.-K., et al., *Appointment waiting times and education level influence the quality of bowel preparation in adult patients undergoing colonoscopy.* BMC gastroenterology, 2011. **11**: p. 1-9.

13. Cheng, P., et al., *3 liters of polyethylene glycol vs. standard bowel preparation have equal efficacy in a Chinese population: a randomized, controlled trial.* American Journal of Translational Research, 2022. **14**(8): p. 5641.

14. Chen, L., et al., *Individualized intervention based on a preparation-related prediction model improves adequacy of bowel preparation: A prospective, multi-center, randomized, controlled study.* Digestive and Liver Disease, 2024. **56**(3): p. 436-443.

15. Chen, S.W., et al., *Optimal procedural sequence for same‐day bidirectional endoscopy with moderate sedation: A prospective randomized study.* Journal of Gastroenterology and Hepatology, 2018. **33**(3): p. 689-695.

16. Choi, H.S., et al., *Orange juice intake reduces patient discomfort and is effective for bowel cleansing with polyethylene glycol during bowel preparation.* Diseases of the Colon & Rectum, 2014. **57**(10): p. 1220-1227.

17. Daniels, G., et al., *A national study comparing the tolerability and effectiveness of colon-cleansing preparations.* Gastroenterology Nursing, 2012. **35**(3): p. 182-191.

18. Denis, B., et al., *Quality assurance and gastrointestinal endoscopy: an audit of 500 colonoscopic procedures.* Gastroentérologie clinique et biologique, 2004. **28**(12): p. 1245-1255.

19. Eckardt, A.J., et al., *Open access colonoscopy in the training setting: which factors affect patient satisfaction and pain?* Endoscopy, 2008. **40**(02): p. 98-105.

20. Fernández-Landa, M.J., et al., *Quality indicators and patient satisfaction in colonoscopy.* Gastroenterología y Hepatología (English Edition), 2019. **42**(2): p. 73-81.

21. Ge, F., et al., *Low-dose of magnesium sulfate solution was not inferior to standard regime of polyethylene glycol for bowel preparation in elderly patients: a randomized, controlled study.* Scandinavian Journal of Gastroenterology, 2023. **58**(1): p. 94-100.

22. Gerard, D.P., et al., *Randomized trial of Gatorade/polyethylene glycol with or without bisacodyl and NuLYTELY for colonoscopy preparation.* Clinical and Translational Gastroenterology, 2012. **3**(6): p. e16.

23. Hamada, Y., et al., *Risk factors associated with painful colonoscopy and prolonged cecal intubation time in female patients.* Journal of the Anus, Rectum and Colon, 2023. **7**(3): p. 168-175.

24. Hori, H., et al., *Efficacy and safety of same‐day preparation with sodium picosulfate plus magnesium citrate on the day of colonoscopy for bowel preparation: Multicenter, single‐arm, open‐label study.* Journal of Gastroenterology and Hepatology, 2024. **39**(10): p. 2151-2157.

25. Jensch, S., et al., *CT colonography with limited bowel preparation: prospective assessment of patient experience and preference in comparison to optical colonoscopy with cathartic bowel preparation.* European radiology, 2010. **20**: p. 146-156.

26. Jung, S.H., et al., *Comparison of 2 L polyethylene glycol plus ascorbic acid and 4 L polyethylene glycol in elderly patients aged 60–79: a prospective randomized study.* Digestive Diseases and Sciences, 2022. **67**(10): p. 4841-4850.

27. Jung, S.W., et al., *Effect of Coffee Added to a Polyethylene glycol plus Ascorbic acid Solution for Bowel Preparation prior to Colonoscopy.* Journal of Gastrointestinal & Liver Diseases, 2016. **25**(1).

28. Jun, J.H., et al., *Randomized clinical trial comparing fixed-time split dosing and split dosing of oral Picosulfate regimen for bowel preparation.* World Journal of Gastroenterology, 2017. **23**(32): p. 5986.

29. Katz, P.O., et al., *A dual-action, low-volume bowel cleanser administered the day before colonoscopy: results from the SEE CLEAR II study.* Official journal of the American College of Gastroenterology| ACG, 2013. **108**(3): p. 401-409.

30. Keane, C., et al., *Comparison of bowel dysfunction between colorectal cancer survivors and a non‐operative non‐cancer control group.* Colorectal Disease, 2020. **22**(7): p. 806-813.

31. Kim, B., et al., *Comparative evaluation of the efficacy of polyethylene glycol with ascorbic acid and an oral sulfate solution in a split method for bowel preparation: a randomized, multicenter phase III clinical trial.* Diseases of the Colon & Rectum, 2017. **60**(4): p. 426-432.

32. Kim, E.S., et al., *A randomized, endoscopist-blinded, prospective trial to compare the preference and efficacy of four bowel-cleansing regimens for colonoscopy.* Scandinavian journal of gastroenterology, 2014. **49**(7): p. 871-877.

33. Kim, S.H., et al., *Combination of bisacodyl suppository and 1 L polyethylene glycol plus ascorbic acid is a non‐inferior and comfortable regimen compared to 2 L polyethylene glycol plus ascorbic acid.* Digestive Endoscopy, 2020. **32**(4): p. 600-607.

34. Kim, Y.S., et al., *Randomized clinical trial comparing reduced-volume oral picosulfate and a prepackaged low-residue diet with 4-liter PEG solution for bowel preparation.* Diseases of the Colon & Rectum, 2014. **57**(4): p. 522-528.

35. Leung, J.W., et al., *A randomized, controlled comparison of warm water infusion in lieu of air insufflation versus air insufflation for aiding colonoscopy insertion in sedated patients undergoing colorectal cancer screening and surveillance.* Gastrointestinal endoscopy, 2009. **70**(3): p. 505-510.

36. Liljegren, A., et al., *Individuals with an increased risk of colorectal cancer: perceived benefits and psychological aspects of surveillance by means of regular colonoscopies.* Journal of clinical oncology, 2004. **22**(9): p. 1736-1742.

37. Lynch, I., et al., *Insufflation using carbon dioxide versus room air during colonoscopy: comparison of patient comfort, recovery time, and nursing resources.* Gastroenterology Nursing, 2015. **38**(3): p. 211-217.

38. Mancini, S., et al., *Preliminary results of a multidisciplinary Italian study adopting a Psycho-Neuro-Endocrine-Immunological (PNEI) approach to the study of colorectal adenomas.* Acta Bio Medica: Atenei Parmensis, 2020. **92**(1): p. e2021014.

39. Manes, G., et al., *Efficacy and acceptability of sodium picosulphate/magnesium citrate vs low‐volume polyethylene glycol plus ascorbic acid for colon cleansing: a randomized controlled trial.* Colorectal Disease, 2013. **15**(9): p. 1145-1153.

40. Manes, G., et al., *Randomized controlled trial comparing efficacy and acceptability of split-and standard-dose sodium picosulfate plus magnesium citrate for bowel cleansing prior to colonoscopy.* Endoscopy, 2014. **46**(08): p. 662-669.

41. Nalankilli, K., et al., *Split‐dose 1 L polyethylene glycol (PEG) with ascorbate is non‐inferior to split‐dose PEG with sodium picosulfate and magnesium citrate with similar tolerability: a randomized study.* Jgh Open, 2021. **5**(9): p. 1026-1032.

42. Nicholson, F.B. and M.G. Korman, *Acceptance of flexible sigmoidoscopy and colonoscopy for screening and surveillance in colorectal cancer prevention.* Journal of medical screening, 2005. **12**(2): p. 89-95.

43. Niv, Y., et al., *Impact of colonoscopy on quality of life.* European journal of gastroenterology & hepatology, 2012. **24**(7): p. 781-786.

44. O'Leary, K.E., et al., *Sex differences in associations between psychosocial factors and aberrant crypt foci among patients at risk for colon cancer.* Gender medicine, 2011. **8**(3): p. 165-171.

45. Parente, F.R., et al., *Overall acceptability and efficacy of commonly used bowel preparations for colonoscopy in Italian clinical practice. A multicentre prospective study.* Digestive and Liver Disease, 2014. **46**(9): p. 795-802.

46. Pohl, J., et al., *Impact of the quality of bowel cleansing on the efficacy of colonic cancer screening: a prospective, randomized, blinded study.* PLoS One, 2015. **10**(5): p. e0126067.

47. Pontone, S., et al., *Low-volume plus ascorbic acid vs high-volume plus simethicone bowel preparation before colonoscopy.* World journal of gastroenterology: WJG, 2011. **17**(42): p. 4689.

48. Pontone, S., et al., *Do difficulties in emotional processing predict procedure pain and shape the patient’s colonoscopy experience?* BMJ open, 2022. **12**(2): p. e050544.

49. Quintana, J.M., et al., *Quality indicators and outcomes in a prospective cohort of colorectal cancer patients.* Journal of Gastrointestinal Cancer, 2023: p. 1-7.

50. Regev, A., et al., *Comparison of Two Bowel Preparations for Colonoscopy: Sodium Picosulphate With Magnesium CitrateVersusSulphate-Free Polyethylene Glycol Lavage Solution.* Official journal of the American College of Gastroenterology| ACG, 1998. **93**(9): p. 1478-1482.

51. Repici, A., et al., *Novel 1-L polyethylene glycol+ ascorbate versus high-volume polyethylene glycol regimen for colonoscopy cleansing: a multicenter, randomized, phase IV study.* Gastrointestinal Endoscopy, 2021. **94**(4): p. 823-831. e9.

52. Rex, D.K., J. McGowan, and J.A. Di Palma, *A randomized, controlled trial of oral sulfate solution plus polyethylene glycol as a bowel preparation for colonoscopy.* Gastrointestinal Endoscopy, 2014. **80**(3): p. 482-491.

53. Ristvedt, S.L., et al., *Patient preferences for CT colonography, conventional colonoscopy, and bowel preparation.* Official journal of the American College of Gastroenterology| ACG, 2003. **98**(3): p. 578-585.

54. Rivas, J.M., et al., *Efficacy of morning-only 4 liter sulfa free polyethylene glycol vs 2 liter polyethylene glycol with ascorbic acid for afternoon colonoscopy.* World Journal of Gastroenterology: WJG, 2014. **20**(30): p. 10620.

55. Rosati, G., et al., *A randomized trial of intensive versus minimal surveillance of patients with resected Dukes B2-C colorectal carcinoma.* Annals of oncology, 2016. **27**(2): p. 274-280.

56. Sadeghi, A., et al., *Low volume polyethylene glycol combined with senna versus high volume polyethylene glycol, which regimen is better for bowel preparation for colonoscopy? A randomized, controlled, and single‐blinded trial.* Health science reports, 2022. **5**(5): p. e829.

57. Samarasena, J.B., et al., *Single-day low-residue diet prior to colonoscopy demonstrates improved bowel preparation quality and patient tolerance over clear liquid diet: a randomized, single-blinded, dual-center trial.* Digestive Diseases and Sciences, 2022. **67**(6): p. 2358-2366.

58. Scaglione, G., et al., *One-Day versus Three-Day Low-Residue Diet and Bowel Preparation Quality before Colonoscopy: A Multicenter, Randomized, Controlled Trial.* Digestive Diseases, 2023. **41**(5): p. 708-718.

59. Seo, E.H., et al., *Low-volume morning-only polyethylene glycol with specially designed test meals versus standard-volume split-dose polyethylene glycol with standard diet for colonoscopy: a prospective, randomized trial.* Digestion, 2013. **88**(2): p. 110-118.

60. Shafer, L., et al., *Factors associated with anxiety about colonoscopy: the preparation, the procedure, and the anticipated findings.* Digestive Diseases and Sciences, 2018. **63**: p. 610-618.

61. Shavakhi, A., et al., *Assessment of pain between sedated and unsedated colonoscopy: double-blind randomized clinical trail.* Journal of Research in Medical Sciences, 2021. **26**(1): p. 36.

62. Sheng, L.-P., et al., *Watching videos of colonoscopies and receiving interpretations reduce pain and anxiety while increasing the satisfaction of patients.* Digestive Diseases and Sciences, 2021. **66**: p. 541-546.

63. Sirinawasatien, A., et al., *Bowel preparation using 2-L split-dose polyethylene glycol regimen plus lubiprostone versus 4-L split-dose polyethylene glycol regimen: a randomized controlled trial.* BMC gastroenterology, 2022. **22**(1): p. 424.

64. Suthadsanavijit, S. and C. Jiraphorncharas, *Comparison between Online and On-Site Pre-Anesthesia Evaluation in Patients Undergoing Colonoscopy: A Randomized Controlled Trial.* Journal of the Medical Association of Thailand, 2022. **105**(4).

65. Tajika, M., et al., *Can mosapride citrate reduce the volume of lavage solution for colonoscopy preparation?* World Journal of Gastroenterology: WJG, 2013. **19**(5): p. 727.

66. Tarr, G.P., et al., *Do high risk patients alter their lifestyle to reduce risk of colorectal cancer?* BMC gastroenterology, 2014. **14**: p. 1-8.

67. Taylor, S.A., et al., *Nonlaxative PET/CT colonography: feasibility, acceptability, and pilot performance in patients at higher risk of colonic neoplasia.* Journal of Nuclear Medicine, 2010. **51**(6): p. 854-861.

68. van Bokhorst, Q.N., et al., *Clinician-reported Gloucester Comfort Scale scores underestimate patient discomfort and pain during colonoscopy: insights from comparison with a patient-reported experience measure.* Endoscopy, 2025. **57**(06): p. 645-657.

69. van Keulen, K.E., et al., *A novel device for intracolonoscopy cleansing of inadequately prepared colonoscopy patients: a feasibility study.* Endoscopy, 2019. **51**(01): p. 85-92.

70. van Liere, E.L., et al., *Colonoscopy surveillance in Lynch syndrome is burdensome and frequently delayed.* Familial Cancer, 2023. **22**(4): p. 403-411.

71. van Vugt van Pinxteren, M.W., et al., *A prospective study of bowel preparation for colonoscopy with polyethylene glycol-electrolyte solution versus sodium phosphate in Lynch syndrome: a randomized trial.* Familial Cancer, 2012. **11**: p. 337-341.

72. Vassallo, R., et al., *Efficacy of 1 L polyethylene glycol plus ascorbate versus 4 L polyethylene glycol in split-dose for colonoscopy cleansing in out and inpatient: A multicentre, randomized trial (OVER 2019).* Digestive and Liver Disease, 2024. **56**(3): p. 495-501.

73. Voiosu, A., et al., *Factors affecting colonoscopy comfort and compliance: a questionnaire based multicenter study.* Rom J Intern Med, 2014. **52**(3): p. 151-157.

74. Atzingen, A.C.v., et al., *Using computed tomography colonography in patients at high risk of colorectal cancer-a prospective study in a university hospital in South America.* Clinics, 2014. **69**(11): p. 723-730.

75. Walker, T.B., et al., *An interactive video educational tool does not improve the quality of bowel preparation for colonoscopy: A randomized controlled study.* Digestive diseases and sciences, 2022. **67**(6): p. 2347-2357.

76. Walter, B., et al., *Smartphone application to reinforce education increases high-quality preparation for colorectal cancer screening colonoscopies in a randomized trial.* Clinical Gastroenterology and Hepatology, 2021. **19**(2): p. 331-338. e5.

77. Walter, B., et al., *Improving the quality and acceptance of colonoscopy preparation by reinforced patient education with short message service: results from a randomized, multicenter study (PERICLES-II).* Gastrointestinal endoscopy, 2019. **89**(3): p. 506-513. e4.

78. Wang, S.-L., et al., *Effect of WeChat and short message service on bowel preparation: an endoscopist-blinded, randomized controlled trial.* European journal of gastroenterology & hepatology, 2019. **31**(2): p. 170-177.

79. Wang, D., et al., *Combined water exchange and cap-assisted colonoscopy reduces cecal intubation time and patient discomfort in patients with unsedated colonoscopy: a randomized controlled trial.* BMC gastroenterology, 2025.

80. Wattchow, D.A., et al., *General practice vs surgical-based follow-up for patients with colon cancer: randomised controlled trial.* British journal of cancer, 2006. **94**(8): p. 1116-1121.

81. Wen, C.-C., S.-W. Jao, and C.-W. Hsiao, *A modified bowel preparation regimen for colonoscopy providing the patients’ satisfaction and convenience.* Medical Science Monitor: International Medical Journal of Experimental and Clinical Research, 2017. **23**: p. 3123.

82. Williams, G., P. Clarke, and K. Vellacott, *Anxieties should not be forgotten when screening relatives of colorectal cancer patients by colonoscopy.* Colorectal Disease, 2006. **8**(9): p. 781-784.

83. Xiong, Z., et al., *2L polyethylene glycol combined with castor oil versus 4L polyethylene glycol for bowel preparation before colonoscopy among inpatients.* Medicine, 2023. **102**(29): p. e34294.

84. Yang, H.J., et al., *Novel sulfate tablet PBK‐1701TC versus oral sulfate solution for colon cleansing: A randomized phase 3 trial.* Journal of Gastroenterology and Hepatology, 2020. **35**(1): p. 29-36.

85. Yang, H.J., et al., *Randomized trial comparing oral sulfate solution with 4‐L polyethylene glycol administered in a split dose as preparation for colonoscopy.* Journal of Gastroenterology and Hepatology, 2017. **32**(1): p. 12-18.

86. Zhang, C., et al., *A novel ultra-low-volume regimen combining 1 L polyethylene glycol and linaclotide versus 2 L polyethylene glycol for colonoscopy cleansing in low-risk individuals: a randomized controlled trial.* Gastrointestinal endoscopy, 2023. **97**(5): p. 952-961. e1.

87. Zhang, S., et al., *Simethicone improves bowel cleansing with low-volume polyethylene glycol: a multicenter randomized trial.* Endoscopy, 2018. **50**(04): p. 412-422.

88. Marmo, R., et al., *Effective bowel cleansing before colonoscopy: a randomized study of split-dosage versus non-split dosage regimens of high-volume versus low-volume polyethylene glycol solutions.* Gastrointestinal endoscopy, 2010. **72**(2): p. 313-320.

89. Fang, J., et al., *Impact of gum chewing on the quality of bowel preparation for colonoscopy: an endoscopist-blinded, randomized controlled trial.* Gastrointestinal Endoscopy, 2017. **86**(1): p. 187-191.

90. Tarr, G.P., et al., *Perceived risks and benefits of surveillance colonoscopy in people undergoing surveillance for family history of colorectal cancer.* The New Zealand Medical Journal (Online), 2013. **126**(1382).

91. Moloney, J., et al., *High efficacy and patient satisfaction with a nurse‐led colorectal cancer surveillance programme with 10‐year follow‐up.* ANZ Journal of Surgery, 2019. **89**(10): p. 1286-1290.
